# Supplementary material for: Retinal Vasculature in Schizophrenia Spectrum Disorder
Source: Bioengineering (Basel). 2025 Dec 28;13(1):35. doi: 10.3390/bioengineering13010035 (PMC12837173; doi:10.3390/bioengineering13010035)
Supplement: Supplementary file 1 [file bioengineering-13-00035-s001.zip › bioengineering-3963691-supplementary.pdf]

bioengineering

MDPI

*Article*

# Retinal Vasculature in Schizophrenia Spectrum Disorder

**Caroline Simon [1], Erik Gunnarsson [1], Nycole Hidalgo [1], Victoria Chen [2], Kevin Zhang [3], Shuo Chen [4,5], Hwiyoung Lee [4,5], Hugh O'Neill [4], L. Elliot Hong [5] and Osamah Saeedi [2,*]**

[1] University of Maryland School of Medicine, Baltimore, MD 21201, USA; caroline.simon@pennmedicine.upenn.edu (C.S.); ekgunnarsson@som.umaryland.edu (E.G.); fhidalgo@som.umaryland.edu (N.H.)
[2] Department of Ophthalmology and Visual Sciences, University of Maryland School of Medicine, Baltimore, MD 21201, USA; victoria.chen@som.umaryland.edu
[3] Department of Pathology, Johns Hopkins University, Baltimore, MD 21218, USA; k.yang.zhang@gmail.com
[4] Division of Biostatistics and Bioinformatics, Department of Epidemiology and Public Health, Baltimore, MD 21201, USA; shuochen@som.umaryland.edu (S.C.); hwiyoung.lee@som.umaryland.edu (H.L.); honeill@som.umaryland.edu (H.O.)
[5] Maryland Psychiatric Research Center, Department of Psychiatry, University of Maryland School of Medicine, Baltimore, MD 21201, USA; ehong@som.umaryland.edu
* Correspondence: author: osaeedi@som.umaryland.edu

## Supplemental Data

For the ONH images, the average age of the included participants was significantly higher than the excluded participants (t=-2.10, p value = 0.041). There were significantly more females in the excluded group compared to the included group (chi-squared = 7.33, p value = 0.007). There was no significant difference in race (chi-squared = 5.27, p value = 0.261), ethnicity (chi-squared = 1.12, p value = 0.290), or diagnosis (chi-squared = 2.23, p value = 0.135) between included and excluded participants in the ONH images. For the macula images, there was no significant difference in age (t = -1.04, p value = 0.302), sex (chi-squared = 0.032, p value = 0.858), race (chi-squared = 3.68, p value = 0.451) or ethnicity (chi-squared = 0.423, p value = 0.516) or diagnosis (chi-squared = 0.084, p value = 0.772) between included and excluded participants. For the FAZ images, there was no significant difference in age (t = -1.49, p value = 0.139), sex (chi-squared = 0.845, p value = 0.358), race (chi-squared = 0.987, p value = 0.912) or ethnicity (chi-squared = 0.178, p value = 0.674) or diagnosis (chi-squared = 0.491, p value = 0.484) between included and excluded participants.

*ONH Vessel Density*

**Table S1.** Average vessel density for whole retina thickness and superficial capillary plexus in the ONH region. Values are reported as mean ± SD. There were no significant differences between groups in any plexus.

| | Control, N = 32 (57 eyes) | SSD, N = 40 (73 eyes) | *p* value |
|---|---|---|---|
| **Whole Retina (WR)** | 15.53 ± 0.83 | 15.67 ± 0.64 | 0.358 |
| **Superficial Capillary Plexus (SCP)** | 14.97 ± 0.61 | 14.88 ± 0.65 | 0.653 |

*Macula Vessel Density*

 https://doi.org/10.3390/bioengineering13010035

**Table S2.** Average vessel density for whole retina thickness and superficial capillary plexus in the macula. Values are reported as mean ± SD. There were no significant differences between groups in any plexus.

| | Control, N = 32 (46 eyes) | SSD, N = 38 (59 eyes) | *p* value |
|---|---|---|---|
| **Whole Retina** | 9.05 ± 0.49 | 9.02 ± 0.59 | 0.868 |
| **Superficial Capillary Plexus** | 8.26 ± 0.51 | 8.20 ± 0.58 | 0.906 |

*Demographics of ONH participants by age group*

**Table S3.** Demographics of participants included in age separate analysis of ONH vessel density.

| **Age </=30 (N=34)** | | | |
|---|---|---|---|
| | **SSD, n = 19** | **Control, n = 15** | **P value** |
| Age, avg +/- SD | 24.18 +/- 3.83 | 23.63 +/- 3.17 | 0.686 |
| Sex, n (%) | | | |
| Male | 14 | 5 | 0.019 |
| Female | 5 | 10 | |
| Race, n (%) | | | 0.081 |
| White | 8 | 8 | |
| Black | 10 | 2 | |
| Asian | 0 | 2 | |
| Native American | 0 | 1 | |
| Other | 1 | 2 | |
| Ethnicity, n (%) | | | 0.863 |
| Non-Hispanic | 18 | 14 | |
| Hispanic | 1 | 1 | |
| **Age >30 (N= 38)** | | | |
| | **SSD, n = 21** | **Control, n = 17** | **P value** |
| Age, avg +/- SD | 41.94 +/- 8.97 | 45.24 +/- 10.16 | 0.295 |
| Sex, n (%) | | | 0.061 |
| Male | 18 | 10 | |
| Female | 3 | 7 | |
| Race, n (%) | | | 0.487 |
| White | 12 | 8 | |
| Black | 8 | 5 | |
| Asian | 0 | 1 | |
| Native American | 0 | 1 | |
| Other | 1 | 2 | |
| Ethnicity, n (%) | | | 0.260 |
| Non-Hispanic | 21 | 16 | |
| Hispanic | 0 | 1 | |

**Table S4.** Correlation Between RNFL Thickness and ONH Vessel Density in HC v SSD: Comparison of p-values Within Each Quadrant. This is an exploratory analysis and was not corrected for multiple comparisons. * Statistically significant *p*-values.

| **Age Group** | | **Superior** | | **Nasal** | | **Inferior** | | **Temporal** | |
|---|---|---|---|---|---|---|---|---|---|
| | | **SSD** | **HC** | **SSD** | **HC** | **SSD** | **HC** | **SSD** | **HC** |
| <30 | Correlation (R) | 0.176 | -0.235 | -0.464 | -0.070 | -0.401 | 0.092 | 0.100 | -0.292 |
| | *p*-value | 0.362 | 0.419 | 0.011 * | 0.811 | 0.031 * | 0.754 | 0.607 | 0.311 |
| >=30 | Correlation (R) | 0.324 | 0.220 | -0.217 | -0.271 | 0.056 | 0.003 | -0.145 | -0.075 |

| | | | | | | | | |
|---|---|---|---|---|---|---|---|---|
| | *p*-value | 0.047* | 0.351 | 0.191 | 0.248 | 0.741 | 0.990 | 0.386 | 0.753 |

**Disclaimer/Publisher's Note:** The statements, opinions and data contained in all publications are solely those of the individual author(s) and contributor(s) and not of MDPI and/or the editor(s). MDPI and/or the editor(s) disclaim responsibility for any injury to people or property resulting from any ideas, methods, instructions or products referred to in the content.
